# The lacewing *Ceraeochrysa caligata* as a potential biological agent for controlling the red palm mite *Raoiella indica*

**DOI:** 10.7717/peerj.7123

**Published:** 2019-06-20

**Authors:** Luis O. Viteri Jumbo, Adenir V. Teodoro, Adriano S. Rêgo, Khalid Haddi, Andréia S. Galvão, Eugênio Eduardo de Oliveira

**Affiliations:** 1 Departamento de Entomologia, Universidade Federal de Viçosa, Viçosa, Minas Gerais, Brasil; 2 Embrapa Tabuleiros Costeiros, Aracaju, Sergipe, Brasil; 3 Programa de Pós-Graduação em Agroecologia, Universidade Estadual do Maranhão, São Luís, Maranhão, Brasil; 4 Instituto Federal do Maranhão, São Luís, Maranhão, Brasil

**Keywords:** Invasive pests, Functional response, Insect predators, Tenuipalpidae, Chrysopidae

## Abstract

**Background:**

Compared to chemical control, the use of naturally occurring biological agents to control invasive pests is less threatening to the environment and human health.

**Objectives:**

Here, we assessed the ability of immature stages of the lacewing *Ceraeochrysa caligata* (Neuroptera: Chrysopidae) to prey upon different developmental stages of the red palm mite *Raoiella indica* (Acari: Tenuipalpidae), one of the most destructive invasive pests of palm trees in Neotropical regions.

**Methods:**

Increasing densities of three stages of *R. indica* (eggs, immature stages, and adult females) were offered to *C. caligata* in coconut leaf arenas. The immature stages of *C. caligata* were less than 24 h old and were starved before being transferring to the arenas. The amount of prey consumed was recorded 6 h after releasing the *C. caligata*.

**Results:**

Our results indicated that the ability of *C. caligata* to feed upon *R. indica* increased with the larval development of the predator. Higher feeding levels and shorter handling times were recorded for the first and second instars of *C. caligata* when preying upon the eggs and immature stages of *R. indica.* Furthermore, *C. caligata* individuals of different stages exhibited differential functional responses according to prey type (i.e., eggs, immatures, or adult females of *R. indica*). *Ceraeochrysa caligata* second instar individuals exhibited a sigmoid increase in consumption rate with increasing prey availability (i.e., a type III functional response) when preying upon immature stages of* R. indica*. However, when preying upon *R. indica* adult females, *C. caligata* second instar individuals exhibited a type II functional response (i.e., an increase in consumption rate with increasing prey availability, before reaching a plateau). Predator individuals of the first and third instar stages exhibited a type II functional response for all prey types.

**Conclusions:**

Collectively, our findings demonstrate that *C. caligata*, especially at the second instar stage, has potential as a tool for ecological management of the red palm mite.

## Introduction

The red palm mite, *Raoiella indica* Hirst (Acari: Tenuipalpidae), is a severe pest of a myriad of ecologically and agriculturally important crops (Carrillo et al., 2012a), especially palm trees (De Assis, De Morais & Gondim, 2013). For instance, these mites have been recorded on 118 plant species that belong to plant families such as Arecaceae, Heliconiaceae, Musaceae, Strelitziaceae, Zingiberaceae, and Pandanaceae (Carrillo et al., 2012a). Native to the Old World, the red palm mite has invaded tropical regions of the New World and is currently distributed in North America (Roda et al., 2012), the Caribbean (Kane & Ochoa, 2006), and South America (Kane et al., 2012; Melo et al., 2018; Navia et al., 2011); there exists a clear and positive correlation between the mite occurrence and the temperature and precipitation levels (Flores-Galano et al., 2017).

*Raoiella indica* Hirst is a multivoltine arthropod species with gregarious behavior, living in colonies of 20–300 individuals that feed on the abaxial leaf surface (Peña et al., 2006). Red palm mite attacks can lead to significant yield reductions in Brazil—the third largest global producer of coconut, *Cocos nucifera* (L.), producing over 6 million tons every year (FAO, 2017) (Peña, Bruin & Sabelis, 2012). The use of synthetic compounds remains the most prevalent strategy to control *R. indica* in coconut plantations (Jayaraj, Natarajan & Ramasubramanian, 1991; Rodrigues & Peña, 2012), despite the need for frequent applications and the risks associated with these control tools (e.g., threats to human health, contamination of the environment, and selection of resistant individuals). However, this control strategy does not show high efficacy against *R. indica* owing to its location on the host plants, which typically limits its exposure to applied acaricides. Furthermore, coconut palm trees may reach over 10 m high, which makes it particularly challenging to control *R. indica* on these hosts through pesticide sprays (Domingos et al., 2013).

The use of naturally occurring biological control agents represents a plausible alternative strategy for controlling *R. indica*. Some studies have shown the potential of several mites and insects capable of preying on *R. indica* (Carrillo et al., 2012b; Hoy, 2012; Peña et al., 2009). Among predatory mites, species of the genus *Amblyseius* (Acari: Phytoseiidae) have attracted attention owing to their abundance throughout the year and ability to feed upon all developmental stages of the red palm mite (Carrillo et al., 2012b; Vásquez & De Moraes, 2013). Among predatory insects, twelve species from five families have been reported preying on *R. indica*, including *Oligota* sp. (Coleoptera: Staphylinidae) (Somchoudhury & Sarkar, 1987), *Aleurodothrips fasciapennis* (Thysanoptera: Phlaeothripidae), and *Ceraeochrysa* sp. (Neuroptera: Chrysopidae) (Peña et al., 2009).

Lacewings (Neuroptera: Chrysopidae) of the genus *Ceraeochrysa* have particular potential as biological control agents of *R. indica* because they have been shown to actively prey upon these mites on coconut plants (Carrillo et al., 2012b). All immature stages of the *Chrysopidae* species feed on small arthropods. The adults show high reproductive capacity (Carvalho & Souza, 2000), high ecological plasticity (Khuhro et al., 2014), and relative tolerance to pesticides (Ono et al., 2017; Pimentel Farias et al., 2018; Rugno, Zanardi & Yamamoto, 2015) and can be readily mass-reared under controlled conditions (López-Arroyo, Tauber & Tauber, 1999). The duration of the developmental period of *C. caligata* immature instars is dependent on temperature and food type, ranging 4–9, 8–9, and 5–16 days for the first, second, and third instars, respectively (Castro et al., 2009; Nogueira et al., 2007; Souza et al., 2014).

The potential of natural enemies as biological control is commonly measured by means of functional response studies. Functional responses evaluate the feeding behavior of predators, which can be influenced by factors such as environmental conditions and the diverse bioecological interactions (e.g., size, behavior, and density) between predators and prey (Aljetlawi, Sparrevik & Leonardsson, 2004; Laws, 2017; Luff, 1983; Solomon, 1949). Three types of functional responses in relation to prey density have been described, including a linear increase (type I), an increase decelerating to a plateau (type II), and a sigmoid increase (type III) (Holling, 1959; Holling, 1966). Predators that exhibit type III functional response show positive density-dependence and are usually regarded as efficient biological control agents (Fernández-arhex & Corley, 2003; Pervez, 2005). However, insects predators frequently exhibit a type II response limited only by the handling time (time required to subdue, consume, and digest the prey) and can be efficient regulators at low prey densities (Munyaneza & Obrycki, 1997; Santos, 1975).

The present study assessed the potential of *C. caligata* (Banks, 1946) as a biological control agent of *R. indica* by conducting functional response bioassays, in which we used predator individuals of all three larval instars preying upon eggs, immatures, and adult females of the red palm mite.

## Material and Methods

### Rearing of predators and collection of red palm mites

Eggs of the lacewing *C. caligata* were collected from abaxial surfaces of coconut leaves infested with the red palm mite *R. indica* at experimental fields of the Embrapa Tabuleiros Costeiros in Aracaju (10°56′46″37°03′12″W), Sergipe State, Brazil. These coconut plants were located in pesticide-free coconut plantations. The eggs were maintained in Petri dishes, and the emerged larvae were kept individually separated during development (i.e., each larva was placed in a separate Petri dish) and fed *ad libitum* with eggs of *Anagasta kuehniella* (Zeller) (Lepidoptera: Pyralidae). Adults were maintained in plastic containers (20 cm in diameter and 30 cm in height) with open tops covered with fine tissue (i.e., organza) for ventilation. Adults were fed *ad libitum* with an artificial diet consisting of a mixture of bee honey and brewer’s yeast (1:1) and provided with cotton wool soaked in distilled water, which was replaced every 2 days (Freitas, 2001). The rearing units were maintained under controlled conditions of temperature (27 ± 2 °C), relative humidity (65 ± 5%), and photoperiod (12:12 L:D). In order to avoid potential field-driven differences among individuals, we used only larvae that came from individuals that remained under the laboratory conditions for at least one generation.

On the day of the experiments, eggs, immature-stage individuals (larvae, protonymphs, and deutonymphs), and adult females of *R. indica* were collected from pesticide-free leaves of coconut plants at experimental fields of the Embrapa Tabuleiros Costeiros.

### Functional response bioassays

The functional responses of the *C. caligata* individuals of three larval instars to *R. indica* eggs, immatures, and adult females were assessed under laboratory conditions, using experimental procedures described by Hassanpour et al. (2009) and Hassanpour et al. (2011). The assay arena consisted of Petri dishes (5 cm in diameter) containing a clean coconut leaflet piece (15 cm^2^) placed upside down on a layer of solidified agar (7 cm^2^ of free area). Immatures stages and adult females of *R. indica* were gently transferred to the coconut leaflet piece using a fine brush, while pieces of leaflets containing eggs were cut and placed in the arena. Surplus eggs were removed to adjust the prey density. The prey densities were 20, 30, 40, 70, 100, and 150 individuals for the first instar of *C. caligata* and 100, 150, 250, 350, 450, and 600 individuals for the second and third instars. The maximum and minimum prey densities for each *C. caligata* instar were determined in preliminary tests. *Ceraeochrysa caligata* larvae (<12 h old) were starved for 12 h before the bioassays. Then, using a brush, *C. caligata* larvae were transferred individually to the experimental arenas with various prey densities. The Petri dishes were covered with perforated Parafilm to prevent the predators from escaping. Predator larvae that did not feed in the first 5 min were excluded from the experiment. The number of prey consumed was recorded 6 h after predator release, and prey were not replaced. Each prey density was replicated 10 times for each *C. caligata* larval instar. The Petri dishes with prey and predators were maintained at 27 ± 2 °C and 65 ± 5% relative humidity with a 12 h scotophase.

### Statistical analyses

The functional responses were estimated by determining the general shape of each functional response curve based on logistic regression of number of prey consumed as a function of mite stage and density using the CATMOD procedure of SAS statistical software (SAS, 2008). The cubic model was initially tested owing to its ability to detect the most possible functional response graph variations (Juliano, 2001), and a polynomial function was fit:

(1) \begin{eqnarray*} \frac{{\mathrm{N}}_{\mathrm{e}}}{{\mathrm{N}}_{0}} = \frac{\exp \nolimits ({\mathrm{P}}_{0}+{\mathrm{P}}_{1}{\mathrm{N}}_{0}+{\mathrm{P}}_{2}{\mathrm{N}}_{0}^{2}+{\mathrm{P}}_{3}{\mathrm{N}}_{0}^{3})}{1+\exp \nolimits ({\mathrm{P}}_{0}+{\mathrm{P}}_{1}{\mathrm{N}}_{0}+{\mathrm{P}}_{2}{\mathrm{N}}_{0}^{2}+{\mathrm{P}}_{3}{\mathrm{N}}_{0}^{3})} ,\end{eqnarray*}

where (N_e_) is the number of mites attacked; (N_0_) is the initial prey density; and P_0_, P_1_, P_2_, and P_3_ are the intercept, linear, quadratic, and cubic coefficients, respectively, associated with the slope of the curve. The signs of P_1_ and P_2_ are used to determine the type of functional response. A significantly negative (P_1_ < 0) linear coefficient indicates that the predator displays a type II functional response, indicating that the proportion of prey consumed declines monotonically with the initial prey density. A significantly positive (P_1_ > 0) linear coefficient indicates that the predator presents a type III functional response (Juliano, 2001).

As our experiments were conducted with prey depletion, we used the random predator equation (Juliano, 2001; Rogers, 1972) to describe the type II and type III functional responses:

(2) \begin{eqnarray*}{\mathrm{N}}_{\mathrm{e}}={\mathrm{N}}_{0}\{1-\exp \nolimits \left[ \alpha \left( {\mathrm{T}}_{\mathrm{h}}{\mathrm{N}}_{\mathrm{e}}-\mathrm{T} \right) \right] \},\end{eqnarray*}

(3) \begin{eqnarray*}{\mathrm{N}}_{\mathrm{e}}={\mathrm{N}}_{0}\{1-\exp \nolimits \left[ \left( \mathrm{d}+\mathrm{b}{\mathrm{N}}_{0} \right) \left( {\mathrm{T}}_{\mathrm{h}}{\mathrm{N}}_{\mathrm{e}}-\mathrm{T} \right) /(1+\mathrm{c}{\mathrm{N}}_{0}) \right] \},\end{eqnarray*}

**Figure 1:**
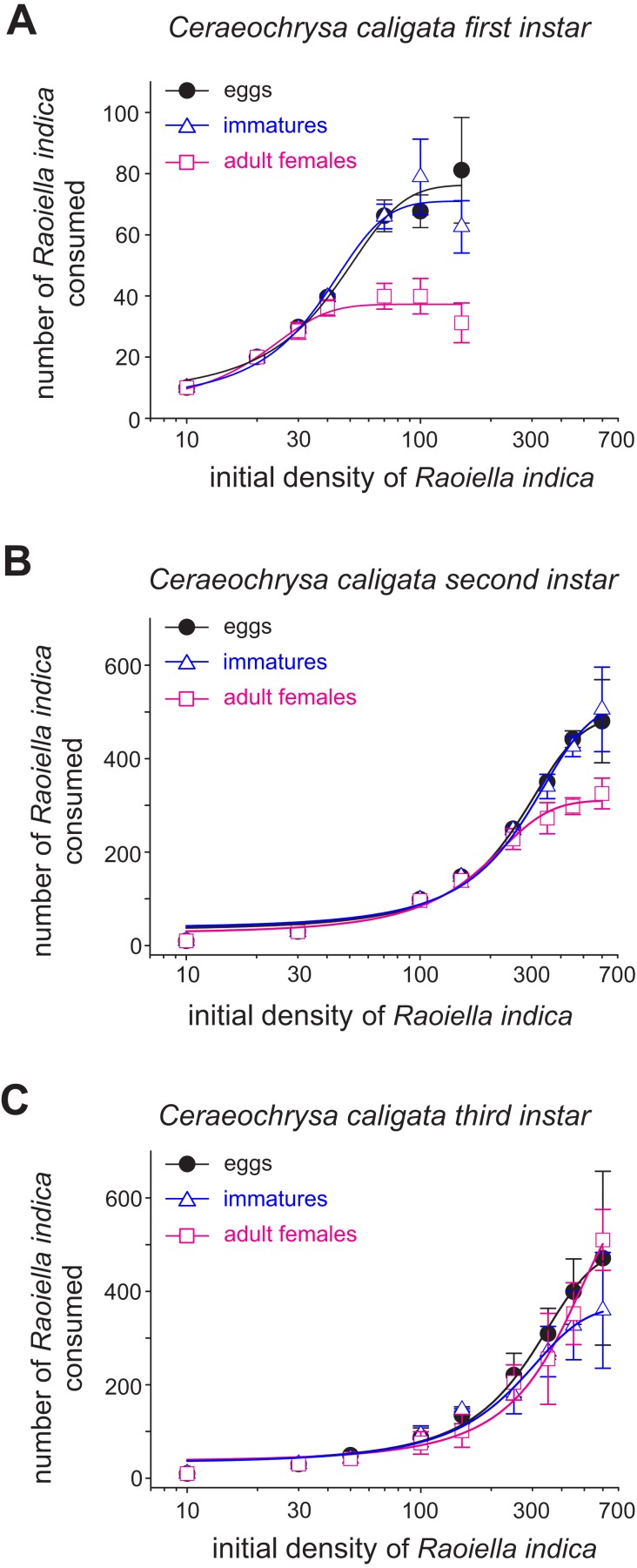
Mean numbers (±SD) of eggs, immatures and females of *R. indica* consumed by first (*A*), second (*B*) and third (*C*) larval instars of the lacewing *Ceraeochrysa caligata* in relation to prey density over a period of 24 h.

where N_e_ is the number of prey attacked; T is the exposure time (6 h); N_0_ is the initial prey density; *α* is the attack rate, a constant rate of successful search; and T_h_ is the handling time. The coefficients *α*, b, c, and d are constants associated with the attack rate. Subsequently, the functional response parameters T_h_ (handling time) and *α* (attack rate) were estimated with nonlinear least square regression using the PROC NLIN procedure of SAS (SAS, 2008) as described elsewhere (Juliano, 2001). Differences in prey consumption among predator larval stages were determined by nonparametric Kruskal–Wallis test (*P* < 0.05).

## Results

**Figure 2:**
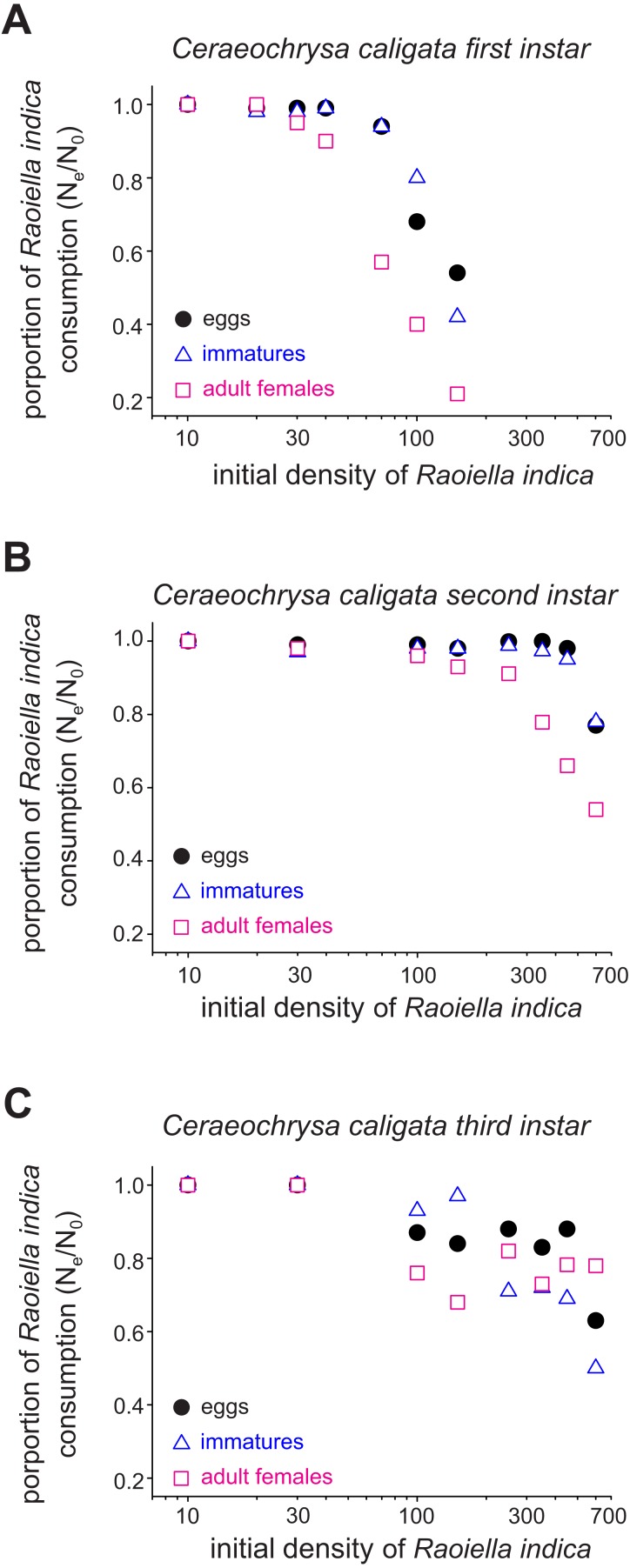
Proportions (Ne/N_o_) of eggs, immatures and females of *Raoiella indica* consumed by first (A), second (B) and third (C) larval instars of *Ceraeochrysa caligata* according to prey density.

The functional response curves for the *C. caligata* individuals of three larval instars indicated that the number of *R. indica* eggs, immatures, or adult females consumed by *C. caligata* rapidly increased with increasing prey density (Fig. 1). Furthermore, whereas logistic regression yielded a significant (*P* < 0.001) negative linear coefficient (i.e., type II functional response) for the first and third instars of *C. caligata* feeding upon all prey stages (Table 1), the second instar individuals of *C. caligata* exhibited a type III functional response (i.e., a positive and statistically significant linear coefficient) when feeding upon eggs and immatures of *R. indica* but a type II functional response when preying upon adult females of *R. indica* (Table 1). A type II functional response was also evident in the proportion of prey consumed (N_e_/N_o_) by first, second, and third instar individuals of the predator (Figs. 2A–2C).

**Figure 3:**
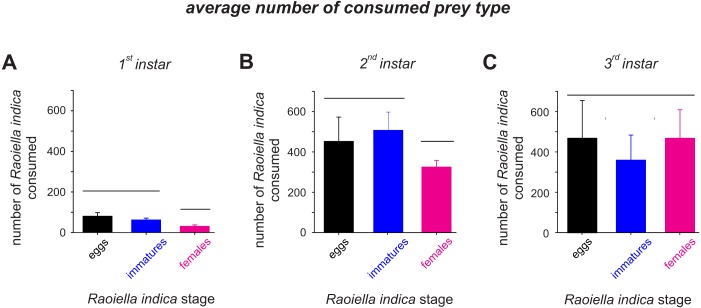
Average consumption (±SD) of eggs, immatures and females of *R. indica* by first (A), second (B) and third larval instars (C) of *Ceraeochrysa caligata*. Bars grouped under the same horizontal line do not differ according to a Kruskal–Wallis test (*P* < 0.05).

The attack rate (*α*) of the *C. caligata* larval instars did not vary by prey type (Table 2). However, the prey handling time in the predator individuals differed significantly with prey type (Table 2). *Ceraeochrysa caligata* individuals of the first and second larval instars exhibited longer handling times with *R. indica* adult females (*First instar*: *T*_*h*_ = 0.146 ± 0.0050, *Second instar*: *T*_*h*_ = 0.018 ± 0.0004) than with the other prey types. In contrast, *C. caligata* individuals of the third larval instar exhibited the shortest handling time with adult females (*T*_*h*_ = 0.010 ± 0.0021) (Table 2). As shown in Fig. 3, the average number of prey consumed (independent of prey type) increased significantly with the developmental stage of the predator. Furthermore, predator individuals of the first (Fig. 3A; *H* = 22.2, *df* = 2, *P* < 0.001) and second (Fig. 3B; *H* = 9.9, *df* = 2, *P* = 0.007) instars consumed significantly fewer adult females than eggs or immatures of *R. indica*. However, no significant differences among prey types were observed in the average number of prey consumed by *C. caligata* individuals of the third larval instar (Fig. 3C; *H* = 3.4, *df* = 2, *P* = 0.188).

## Discussion

Functional responses are well-established parameters used to estimate the potential use of natural enemies as biological agents for controlling arthropod pests (Cuthbert et al., 2018; Ebrahimifar, Jamshidnia & Allahyari, 2017; Milonas, Kontodimas & Martinou, 2011; Solomon, 1949). Here, we demonstrated that all larval instars of the lacewing *C. caligata* are capable of preying upon all developmental stages (i.e., eggs, immatures, and adults) of the red palm mite *R. indica*. Our findings revealed that although immature individuals of *C. caligata* chiefly exhibited type II functional responses (which are highly relevant when prey densities are low), second instar individuals of the predator might be more capable of suppressing *R. indica* populations, because they exhibited a type III functional response when preying upon eggs and immature individuals of *R. indica*.

**Table 1:** Estimated parameters of the logistic regression of the proportion of eggs, immatures and females of *Raoiella indica* consumed by the first, second and third larval instars of *Ceraeochrysa caligata*.

**Prey stage**	**Parameter**	**Larval instar of***Ceraeochrysa caligata*											
		**1**^**st**^				**2**^**nd**^				**3**^**rd**^			
		**Estimate**	**SD**	***x***^**2**^	***p***	**Estimate**	**SD**	***x***^**2**^	***p***	**Estimate**	**SD**	***x***^**2**^	***p***
Egg	Intercept (P_0_)	10.883	0.857	161.5	<0.0001	2.328	0.366	40.50	<0.0001	3.351	0.230	211.34	<0.0001
	**Linear (P**_**1**_**)**	**−0.160**	**0.015**	**109.3**	**<0.0001**	**0.023**	**0.002**	**120.4**	**<0.0001**	**−0.015**	**0.002**	**34.33**	**<0.0001**
	Quadratic (P_2_)	6 ×10^−4^	7 ×10^−5^	82.90	<0.0001	−4.0 ×10^−6^	2.0 ×10^−7^	260.4	<0.0001	4.8 ×10^−5^	7.7 ×10^−6^	38.99	<0.0001
	Cubic (P_3_)	6.8 ×10^−6^	2.3 ×10^−9^	8.70	0.0033	6.0 ×10^−8^	2.9 ×10^−8^	4.10	0.0418	−4.9 ×10^−8^	7.1 ×10^−9^	48.3	<0.0001
Immature	Intercept (P_0_)	5.729	0.505	128.68	<0.0001	−3.545	0.859	17.05	<0.0001	4.057	0.213	364.01	<0.0001
	**Linear (P**_**1**_**)**	**−0.049**	**0.009**	**26.11**	**<0.0001**	**0.099**	**0.011**	**89.46**	**<0.0001**	**−0.024**	**0.002**	**123.88**	**<0.0001**
	Quadratic (P_2_)	6 ×10^−5^	4.3 ×10^−5^	1.97	0.1605	−3 ×10^−4^	3.2 ×10^−5^	90.05	<0.0001	6.3 ×10^−5^	6.5 ×10^−6^	94.23	<0.0001
	Cubic (P_3_)	4.7 ×10^−6^	1.8 ×10^−6^	6,60	0.0102	2.3 ×10^−7^	2.8 ×10^−8^	81.69	<0.0001	−5.6 ×10^−8^	5.8 ×10^−9^	88.84	<0.0001
Adult female	Intercept (P_0_)	8.387	0.774	117,42	<0.0001	4.509	0.167	726.84	<0.0001	1.796	0.148	148.30	<0.0001
	**Linear (P**_**1**_**)**	**−0.222**	**0.030**	**56,78**	**<0.0001**	**−0.012**	**8.1 ×10**^−4^	**216,74**	**<0.0001**	**−0.009**	**0.002**	**29.85**	**<0.0001**
	Quadratic (P_2_)	0.002	0.001	31,87	<0.0001	7 ×10^−6^	9 ×10^−8^	70.60	<0.0001	2.7 ×10^−5^	5.3 ×10^−6^	25.84	<0.0001
	Cubic (P_3_)	−5.9 ×10^−6^	1.2 ×10^−6^	23,84	<0.0001	2.9 ×10^−9^	7.9 ×10^−9^	14.43	0.0001	−2.2 ×10^−8^	4.9 ×10^−9^	19.86	<0.0001

**Notes.**

Negative and positive linear terms (P_1_) denote type II and III functional responses, respectively.

Although three types of functional responses may be exhibited by predators (Holling, 1959), type II is the most common in insects (Begon, Harper & Townsend, 1999), including chrysopids (Montoya-Alvarez et al., 2010; Parajulee et al., 2006; Stewart, Braman & Pendley, 2002). The type II functional response is characterized by an increase in consumption rate with increasing prey availability until reaching a plateau at which the consumption rate stabilizes (Holling, 1959), yielding a negative value for the linear parameter (Juliano, 2001). This kind of functional response is generally limited only by handling time, which allows the predator to effectively control the prey population when prey density is low (Munyaneza & Obrycki, 1997; Santos, 1975). However, some mites and insect predators exhibit type III functional responses; in theory, such predators are more efficient than those with type II responses in suppressing prey populations in biological control programs (Holling, 1966; Huffaker, Messenger & DeBach, 1971), because they exhibit positive density-dependent behaviors (Fernández-arhex & Corley, 2003; Pervez, 2005).

In the present investigation, as previously demonstrated for other lacewing species (Hassanpour et al., 2011; Hassanpour et al., 2009; Sultan & Khan, 2014), immature-stage individuals of the lacewing *C. caligata* chiefly showed type II functional responses. Second instar *C. caligata* individuals preying upon eggs and immatures of *R. indica* showed type III responses. Variation in functional response type can be partially explained by several factors including variation in the size and density of both prey and predator (Aljetlawi, Sparrevik & Leonardsson, 2004; González-Suárez et al., 2011; Hassanpour et al., 2015; Hassanpour et al., 2011; Hassell, Lawton & Beddington, 1977; Kabissa et al., 1996; Milonas, Kontodimas & Martinou, 2011; Sultan & Khan, 2014). It is known that body size plays a crucial role in predator–prey interactions (Aljetlawi, Sparrevik & Leonardsson, 2004; Thorp et al., 2018). For instance, first instars of insect predators face more difficulties in preying upon large prey, and more developed instars exhibit higher predatory abilities when feeding on smaller prey, indicating important consequences for the dynamics of prey–predator systems (De Roos, Persson & McCauley, 2003; Nordlund & Morrison, 1990). Therefore, it is reasonable to argue that second instar individuals of *C. caligata* would be more capable of suppressing *R. indica* populations than would *C. caligata* at other stages, owing to their greater ability to control this mite at the egg and immature stages. However, the potential of the other larval instars of *C. caligata*, for which type II functional responses were observed, should not be dismissed, because first and second instar larvae of *C. caligata* can efficiently control red palm mite populations when these invasive pests are at low densities.

**Table 2:** Attack rate (*α*) and handling time (*T*_*h*_) of the first, second and third larval instars of *Ceraeochrysa caligata* feeding upon eggs, immatures and adult females of *Raoiella indica.*

**Predator stage**	**Prey stage**	**Parameter**						
		**Attack rate(*α*)**	**Asymptotic 95% CI**		**Handling time(T**_**h**_**)**	**Asymptotic 95% CI**		*R*^**2**^
			***Lower***	**Upper**		***Lower***	**Upper**	
1st	Egg	0.032 ± 0.0108a	0.0107	0.0539	0.076 ± 0.0025b	0.071	0.0810	0.98
	Immature	0.050 ± 0.0283a	−0.0067	0.1067	0.082 ± 0.0032b	0.075	0.0879	0.94
	Adult Female	0.021 ± 0.0023a	0.0158	0.0252	0.146 ± 0.0050a	0.136	0.1561	0.82
2nd	Egg	0.023 ± 0.0366a	−0.0499	0.0965	0.013 ± 0.0007a	0.011	0.0141	0.99
	Immature	0.006 ± 0.0018a	0.0020	0.0090	0.010 ± 0.0005a	0.009	0.0120	0.99
	Adult Female	0.005 ± 0.0007a	0.0034	0.0063	0.018 ± 0.0004a	0.017	0.0189	0.99
3rd	Egg	0.003 ± 0.0009a	0.0011	0.0047	0.011 ± 0.0011a	0.009	0.0163	0.98
	Immature	0.003 ± 0.0007a	0.0013	0.0041	0.016 ± 0.0010a	0.013	0.0176	0.97
	Adult Female	0.001 ± 0.0003a	0.0006	0.0017	0.008 ± 0.0016a	0.005	0.0120	0.98

**Notes.**

Values within columns followed by the same letter are not significantly different as determined by confidence interval (CI ±95%).

A valuable indicator of consumption rate and predator efficacy is handling time, which is defined as the cumulative time invested in capturing, killing, and digesting prey (Veeravel & Baskaran, 1997). Attack rate is another relevant parameter when considering potential biological agents and indicates the capture success of the predator, which is influenced by prey size as well as by processes such as searching, detecting, and encountering prey (Ball et al., 2015; Holling, 1959). In the present study, prey type did not affect the attack rates of *C. caligata* individuals of each instar. However, the attack rate was higher in the first instar *C. caligata* individuals than in individuals of the other instars, which suggests that if *C. caligata* initially prefers to feed upon prey of smaller size (i.e., *R. indica* eggs and immature-stage individuals), this feeding preference is reversed as the predator developmentally advances. Prey size has been reported to influence the feeding preferences of other lacewings (Aqueel et al., 2014; Nordlund & Morrison, 1990). For instance, Nordlund & Morrison (1990) reported that individuals of the last larval instar of *Chrysoperla rufilabris* preferentially fed on larger prey when they were offered a choice between *Heliothis virescens* caterpillars and the aphid *Aphis gossypii*.

The *C. caligata* second instar larvae exhibited higher consumption of *R. indica* eggs and immatures than did the *C. caligata* first instar larvae, but prey consumption did not differ between the second and third instar larvae of the predator. Although some studies have shown no increase in prey consumption rate as the predator developed (Atlıhan, Kaydan & Özgökçe, 2004; Chen & Liu, 2001; Fonseca, Carvalho & Souza, 2000; Huang & Enkegaard, 2010), our findings are consistent with the results of some other studies (Jose-Pablo et al., 2017; Sultan & Khan, 2014). The buccal apparatus of *C. caligata* is equipped with a sclerotized, elongate, acutely pointed, and serrated jaw (McEwen, New & Whittington, 2007) that can easily penetrate *R. indica* of all developmental stages. However, eggs and immatures of *R. indica* may have been easily preyed upon because such individuals are immobile (eggs) or exhibit low mobility relative to adult *R. indica* females. Alternatively, the higher consumption of *R. indica* eggs and immatures than of adult *R*. *indica* by *C. caligata* may be related to the differences in biomass and nutrient content among the developmental stages of *R. indica*. Because the eggs and immature-stage individuals of *R. indica* have a lower biomass than that of the adults and may have dissimilar nutrient constitutions, *C. caligata* individuals of the second larval instar may have had to increase their consumption of eggs and immatures to overcome these difficulties.

Although not addressed in the present study, the absence of difference between the feeding capacities of the second and third instar larvae of *C. caligata* may have resulted from an absence of significant differences between these stages in predator voracity, energy storage needs, locomotor ability, or prey-handling efficiency (Atlıhan, Kaydan & Özgökçe, 2004; Bressendorff & Toft, 2011; Hassanpour et al., 2011; McEwen, New & Whittington, 2007; Schmidt et al., 2012). Further investigations are also required to test the prey preference (e.g., eggs, immatures, or adults of *R. indica*) and the survival rates of each predator larval instar when they were fed each *R. indica* developmental phase. In terms of field application, which was not the main point of the present investigation, it will be worthwhile to first evaluate not only the best time of introduction and density ranges for the *C. caligata* larvae but also the agricultural procedures (e.g., plant fertilization and pesticide applications) that are compatible with the naturally occurring arthropods (e.g., insect predators) that provides ecological services.

## Conclusions

The findings described in the present investigation indicate that *C. caligata* has potential as a biological agent to control all stages of the red palm mite. The functional response results suggest that the second and third instar larvae of *C. caligata* are more efficient regulators of high- and low-density red palm mite populations, respectively. The high rates of consumption of *R. indica* eggs and immatures by the *C. caligata* third instars can prevent the emergence and reproduction of new *R. indica* individuals.

##  Supplemental Information

10.7717/peerj.7123/supp-1 Supplemental Information 1 Raw data collected for functional response of *C. caligata* (lacewing: first, second and third instars) feeding on different stages (egg, immature and adult) of *R. indica* (red palm mite)

## Additional Information and Declarations

### Data Availability

The following information was supplied regarding data availability: Raw data are available in a Supplemental File.
